# A Rare Case of Epithelioid Malignant Peripheral Nerve Sheath Tumor Mimicking Malignant Melanoma

**DOI:** 10.7759/cureus.13424

**Published:** 2021-02-18

**Authors:** Layana R Biglow, Jonathan Cuda, Jennifer Dotson

**Affiliations:** 1 Internal Medicine, Marshall University Joan C. Edwards School of Medicine, Huntington, USA; 2 Pathology, Marshall University Joan C. Edwards School of Medicine, Huntington, USA; 3 Oncology, Marshall University Joan C. Edwards School of Medicine, Huntington, USA

**Keywords:** malignant peripheral nerve sheath tumour, soft tissue neoplasm, desmoplastic melanoma

## Abstract

Malignant peripheral nerve sheath tumors are rare soft tissue sarcomas that are often associated with neurofibromatosis type-1. These tumors share common immunohistochemistry findings which can make diagnosis difficult. We present the case of a woman who presented with a diagnosis of metastatic melanoma of the index finger of her left hand but was eventually diagnosed with primary epithelioid malignant peripheral nerve sheath tumor. We will be highlighting the diagnostic challenge of differentiating between these two very different malignancies.

## Introduction

Malignant peripheral nerve sheath tumors (MPNSTs) are rare soft tissue neoplasms that usually arise from peripheral nerves and show differentiation toward one of the cellular components of the nerve sheath including Schwann cells, fibroblasts, and perineural cells. [1] These neoplasms can arise sporadically or may be associated with patients who have neurofibromatosis-type 1 (NF1). The latter may arise either de novo or from a pre-existing neurofibroma. Also, approximately 10% of tumors are associated with previous radiation exposure and considered to be indolent malignancies [1]. Malignant peripheral nerve sheath tumors arise most often in the extremities, particularly proximally, followed by the trunk, head, and neck. Clinical presentation includes the development of a painful and/or rapidly enlarging mass with associated neurological deficits. The biological behavior of MPNSTs has been described as unpredictable in previous research and the following are predictors of locoregional recurrence and distant metastasis: anatomic site of tumor, larger tumors larger than 10 cm, adequacy of resection margins, American Joint Committee on Cancer (AJCC) stage III, lack of S100 staining, elevated Ki67 expression (more than 25%) as well as nuclear tumor protein p53 (TP53) and mouse double minute 2 (MDM2) expression [2]. The recurrence rate after definitive treatment may be up to 40 percent and approximately 66 percent may metastasize hematogenously to the lungs and bone. Epithelioid MPNSTs, which are defined as greater than 50 percent of polygonal tumor cells in sheets or nodules, are an exceedingly rare variant of MPNSTs accounting for only 5 percent of all cases. It has distinct immunophenotypical characteristics [2]. Several tumors can mimic MPNSTs, most importantly spindle cell/desmoplastic melanoma, which serves as the main differential due to the higher incidence, remarkably similar morphology and overlapping immunochemical markers [3]. Radical resection remains the primary therapy for MPNSTs due to its limited sensitivity to chemotherapy and radiotherapy [1]. The distinction between melanoma and MPNSTs presents a diagnostic challenge due to radical differences in therapeutic implications and therefore it is crucial to recognize this possibility during pathologic review [2]. In this case, we present a 47-year-old female diagnosed with a primary epithelioid malignant peripheral sheath tumor mimicking malignant melanoma.

## Case presentation

**Figure 1:**
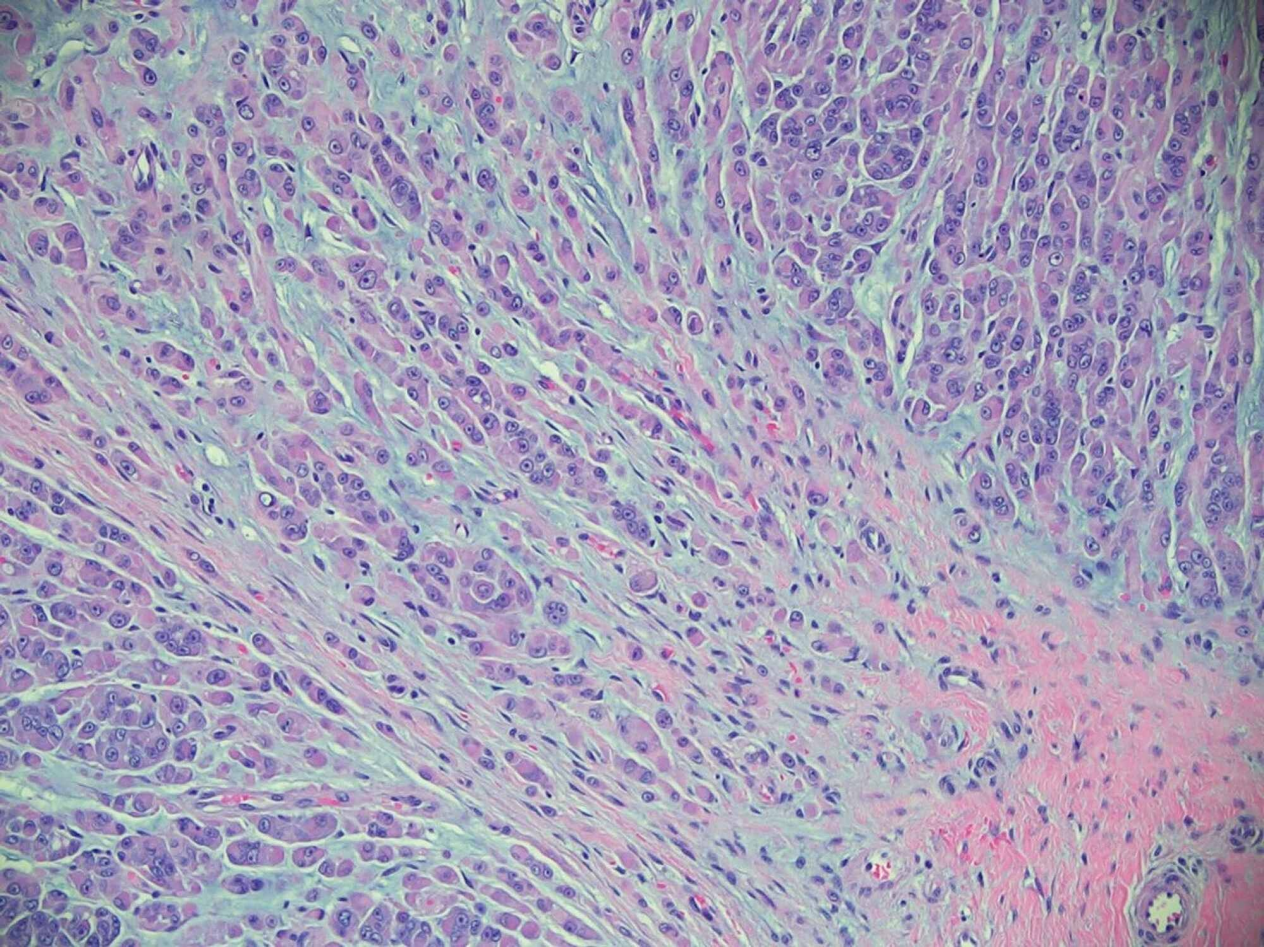
Epithelioid malignant peripheral nerve sheath tumor, hematoxylin, and eosin stain 200X. Subcutaneous tissue with malignant epithelioid tumor cells displaying prominent nucleoli, eosinophilic cytoplasm, and myxoid stroma. Tumor cells were immunoreactive for S100 and Sox-10 with loss of integrase interactor-1, or INI-1 (not shown).

A 47-year-old female presented to our outpatient oncology clinic as a referral by her primary care provider for a recent diagnosis of melanoma of the finger. The patient had noticed a subcutaneous nodule without any obvious nevus or skin color changes, which arose 12 months prior to presentation with gradual increase in size to about one to two centimeters. The patient described the initial nodule as semi-solid. Her only past medical history consisted of a benign renal tumor and right nephrectomy and osteoarthritis. She was a current smoker but denied frequent sun exposure. Her primary care provider performed an excision of the nodule, which the patient described as a white semi-solid mass. Initial pathologic review revealed an epidermal inclusion cyst. The specimen was then sent for additional pathologic review. Their report described the specimen as “malignant melanoma,” and likely a metastatic deposit. The report described a well-circumcised epithelioid neoplasm with surrounding fibrosis and a myxoid appearance (Figure 1). The neoplastic cells appeared to be epithelioid with vascular nuclei and prominent nucleoli with scattered mitotic figures. Immunohistochemistry (IHC) was positive for the S100 protein, Sry-related HMg-Box gene 10 (SOX10), B-cell lymphoma 2 (BCL-2), vimentin, focally positive for smooth-muscle actin (SMA), and weakly positive for phosphoprotein 53 (p53). The IHC stains were negative for tumor protein p63, cytokeratin, melan-A, human melanoma black-45 (HMB-45), anti-melanoma antibody (PNL-2), B-Raf proto-oncogene (BRAF), epithelial membrane (EMA), CD34, desmin and carcinoembryonic antigen (CEA). The mass showed that 5 to 10 percent of cells were positive for Ki-67, and 15-20 percent of cells were positive for programmed death-cell ligand (PD-L1).

**Figure 2:**
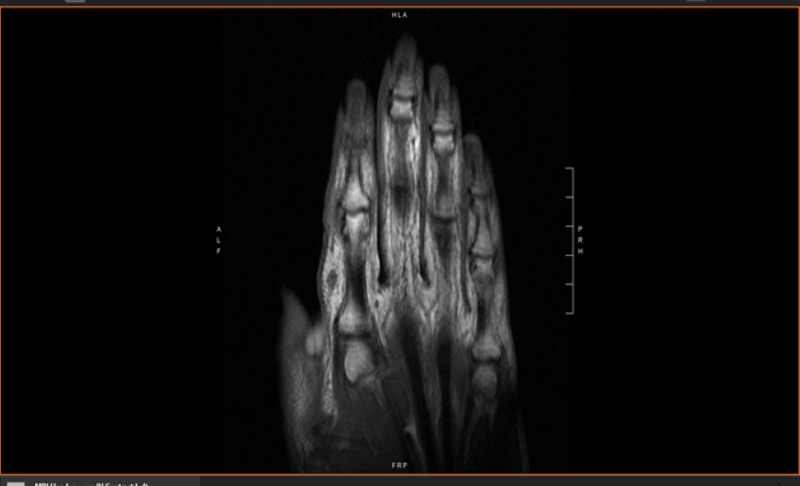
Magnetic resonance imaging of left hand showing approximate 1 cm subcutaneous mass on lateral aspect of index finger.

**Figure 3:**
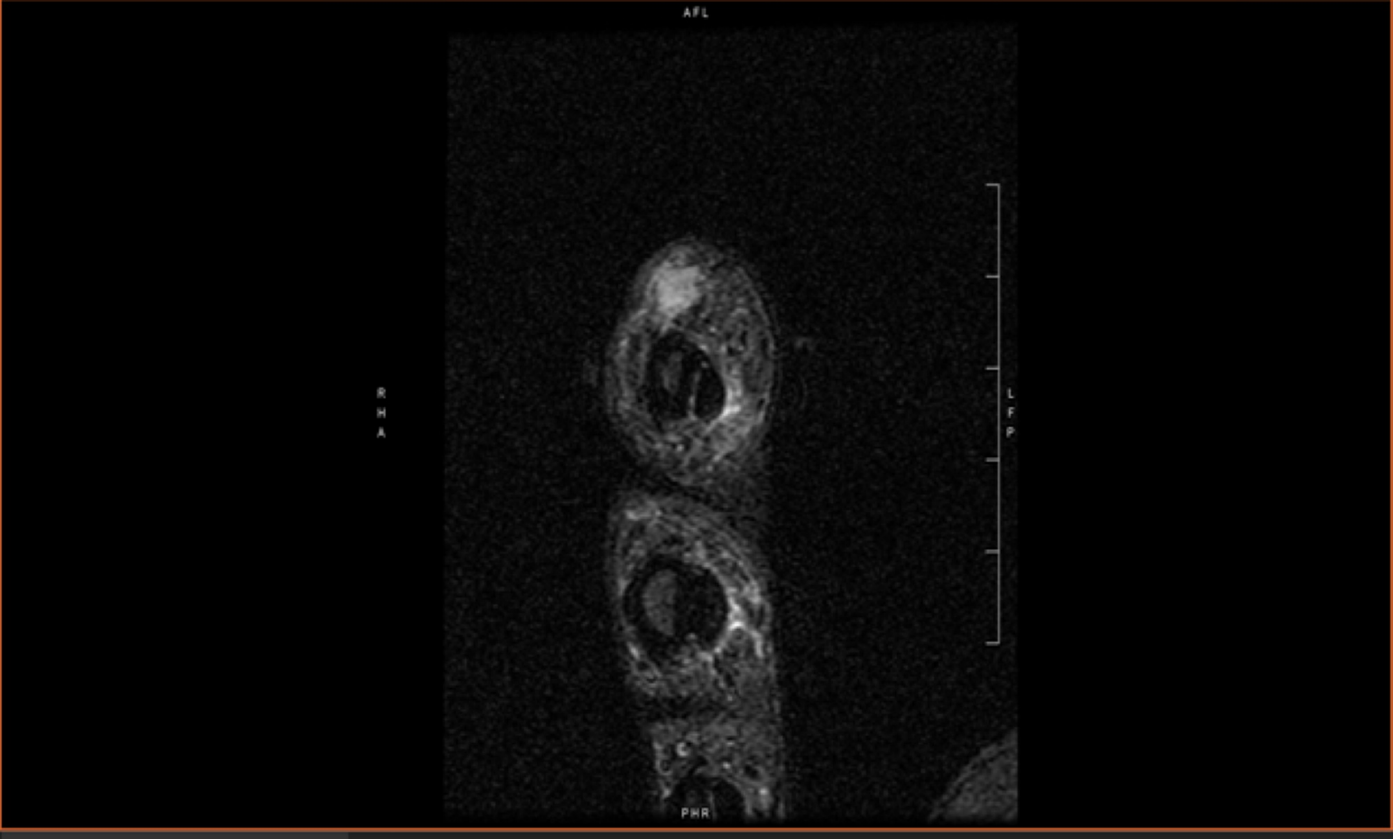
Magnetic resonance imaging of left hand showing approximate 1 cm subcutaneous mass on lateral aspect of index finger.

The patient had a full skin examination by dermatology which did not reveal any concerning skin lesions besides a few subcentimeter benign appearing nevi on the lower extremities. Computed tomography (CT) scan of the chest, abdomen and pelvis showed a three millimeter (mm) lung nodule, a small hemangioma of the liver and no other concerning findings for metastatic disease. On presentation to the oncology clinic, physical examination was significant for healing incision at the base of the left second finger. Since the excision, the patient now had a recurrent subcutaneous nodule measuring one centimeter (cm) in size. The nodule was mobile and semi-solid with no associated skin color changes or nevi. Subsequently, the patient had a positron emission tomography f-18 fluorodeoxyglucose and computed tomography scan (f-18 FDG PET/CT) which showed no hypermetabolic lesions. Due to the unusual presentation of an unpigmented subcutaneous mass with no pertinent skin lesions or metastatic foci, we chose to do an additional pathologic review. A joint review by our institution and Johns Hopkins, additional immunostains showed expression of SOX10 again, as well as a loss of integrase interactor 1 (INI-1). Expression of Ewing sarcoma breakpoint region 1 (ESWR1) by fluorescence in-situ hybridization was negative. The above findings were consistent with epithelioid type MPNST. Additional magnetic resonance imaging (MRI) of the left hand revealed a 0.8 x 0.5 x 0.8 cm subcutaneous mass on the lateral aspect of the index finger at the level of the midportion of the proximal phalanx. This lesion showed intermediate signal intensity similar to muscle on T1-weighted sequences and hyperintense on short tau inversion recovery (STIR) sequence; it also showed strong uniform enhancement with no other soft tissue lesions on the visualized portion of digits (Figures 2 and 3).

The patient underwent definitive oncologic surgery with radical resection of soft tissue tumor on the left hand with wound repair and closure, which had negative margins. No radiotherapy or chemotherapy was recommended. The patient remains with no local recurrence over twelve months from surgery.

## Discussion

Malignant peripheral nerve sheath tumors (MPNST) are malignant neoplasms accounting for 3 to 10 percent of soft tissue sarcomas and arise from peripheral nerves or from extra-neural soft tissue. The diagnosis of MPNST continues to be challenging and problematic despite improvement in immunohistochemical and molecular diagnostic tools for sarcomas. [3]. The pathology of this patient’s tumor was described as an epithelioid neoplasm with surrounding fibrosis, with myxoid appearance and scattered mitotic figures (Figure 1), which is consistent with MPNST histology described in the literature. Morphologically, low-to-intermediate grade cutaneous MPNST exhibit a spindle cellular proliferation with nuclear crowding and moderate atypia while high-grade cutaneous MPNST also exhibit alternating hypocellular and hypercellular fascicles of spindle cells with areas of geographic necrosis [4]. Based on this description, our patient’s lesion can be described as high grade.

The cutaneous presentation of MPNST is highly irregular and must be excluded from other tumors such as malignant melanoma, spindle cell squamous cell carcinoma, cutaneous clear cell sarcomas, leiomyosarcoma, and atypical fibroxanthoma [5]. Immunohistochemical staining can be the key in the differential diagnosis of malignant melanoma and MPNST. Historically, histological markers such as the S100 protein and SOX10 have been expressed in both malignancies. However, there is a distinction in distribution that is diagnostically relevant: malignant melanoma is traditionally diffusely and strongly positive for S100, while MPNSTs are only focally positive for this marker. Other markers used to differentiate between spindle/desmoplastic malignant melanoma and MPNST are HMB45, tyrosinase, melanoma-associated antigen (NKI-C3), melanoma antigen recognized by T cells (MART1 or melan-A), and microphthalmia transcription factor (MITF), but research shows there is low sensitivity for these markers in malignant melanoma [6]. The immunohistologic staining of our patient’s neoplasm showed expression of S100, SOX10 and was negative for melan-A, and HMB45 (Figure 1), which fits the profile of MPNST but not specific enough to differentiate from malignant melanoma. Therefore, loss of expression of INI-1 was the discriminating finding in this patient’s case.

Epithelioid MPNST is an uncommon histological variant of MPNSTs which accounts for approximately 5 percent of soft-tissue sarcoma cases and presents predominantly in superficial or deep soft tissues of the extremities. These tumors typically consist of vague nodules of cords, strands, or clusters of large rounded cells with prominent nucleoli. Most epithelioid MPNST strongly and diffusely expresses S100, which contrasts with conventional MPNST, but lack classical melanoma-associated markers such as HMB45. INI-1/SMARCB1 (SWI/SNF-related matrix-associated actin-dependent regulator of chromatin subfamily B member 1) is a tumor suppressor gene at chromosomal band 22q11.2 that encodes a member of the SW1/SNF chromatin remodeling complex and acts as a negative regulator of the cell cycle, is lost in half of the epithelioid MPNSTs. It is also lost in 90 percent of epithelioid sarcomas and malignant extrarenal rhabdoid tumors and retained in desmoplastic melanoma [1]. Epithelioid MPNST in superficial soft tissues is associated with a more favorable course than deep tumors with a metastatic rate of only 12 percent as opposed to 30 percent, respectively [1]. With regards to oncological management and prognosis of desmoplastic melanoma and MPNST, a correct diagnosis is crucial to prevent unnecessary and invasive procedures. Both neoplasms require a wide excision with 1-2 cm margins, but conventional melanoma requires an additional sentinel lymph node biopsy procedure for staging and treatment planning. Furthermore, radiation is sometimes used as an adjunct therapy to surgical excision in MPNST as it has been shown to reduce recurrence and improve local tumor control, though it does not improve overall survival.

In addition, literature review on the subject of MPNSTs reveals additional attempts at identifying markers that may increase specificity or sensitivity for this rare neoplasm. In a multicentered research study conducted in August 2018, researchers created a genome-wide methylation array including probes targeting 866,562 CpG islands to find a methylome signature that could distinguish cutaneous MPNST from spindle/desmoplastic melanoma. They found that branch-chained aminotransferase-1 (BCAT1), an essential enzyme in the leucine catabolic pathway that promotes tumor growth through activation of the mammalian target of rapamycin promoter (mTOR), showed high methylation in the spindle/desmoplastic melanoma group but was hypomethylated in the cutaneous MPNST group. It was then concluded that a unique genome-wide methylome signature for differentiation between spindle/desmoplastic and cutaneous MPNST, and a study on BCAT1 expression as a potential immunohistochemical biomarker for cutaneous MPNST is currently ongoing [4].

In another study, Gaspard et al. investigated the expression of eight melanocytic markers in a cohort of primary MPNST. The study found that there was an expression of certain markers previously thought to be specific to melanoma. They found that 14 percent of MPNST expressed melan-A; 6 percent of MPNST were positive for tyrosinase and 8 percent expressed immunostaining for MITF, which led to the conclusion that these markers were not specific for melanoma. In conclusion, this study found that 20 percent of MPNST, including more than 60 percent of NF1-associated cases, showed diffuse immunostaining with melan-A. This could lead to misdiagnosis and pathologists should be aware that this marker is not genetically specific for melanoma [3].

## Conclusions

In conclusion, definitive distinction between malignant melanoma and malignant peripheral nerve sheath tumors is essential due to differences in treatment and prognosis. Progress has been made to easily distinguish MPNST from its common mimic, desmoplastic melanoma, and ongoing research has made great strides in finding a biomarker for this extremely rare type of neoplasm.
